# Tetra­pyrazine­platinum(II) bis­(tetra­fluoro­borate) acetonitrile hemisolvate

**DOI:** 10.1107/S1600536808033679

**Published:** 2008-10-22

**Authors:** Paul J. Derry, Xiaoping Wang, Bradley W. Smucker

**Affiliations:** a Department of Chemistry, Austin College, 900 North Grand, Sherman, TX 75090-4400, USA; b Oak Ridge National Laboratory, PO Box 2008 MS6460, Oak Ridge, TN 37831-6460, USA

## Abstract

The improved synthesis and characterization of tetra­pyrazine­platinum(II) bis­(tetra­fluoro­borate) acetonitrile hemisolvate, [Pt(C_4_H_4_N_2_)_4_](BF_4_)_2_·0.5CH_3_CN, is reported. The unit cell contains a half equivalent of an acetonitrile solvent mol­ecule per tetra­pyrazine­platinum(II) ion. The coordination geometry of the Pt^II^ ion is almost square-planar, with the Pt atom residing on an inversion center. The BF_4_^−^ counter-anion, located at a general position, has an idealized tetra­hedral geometry and an acetonitrile solvent mol­ecule, the methyl group of which is disordered over two equal positions, sits on a twofold rotation axis.

## Related literature

For general background, see: Derossi *et al.* (2007 ▶); Klika *et al.* (2007 ▶); Pearson *et al.* (1960 ▶); Schweiger *et al.* (2001 ▶); Wendt *et al.* (1997 ▶); Willermann *et al.* (2006 ▶). For related structures, see: Wei *et al.* (1989 ▶).
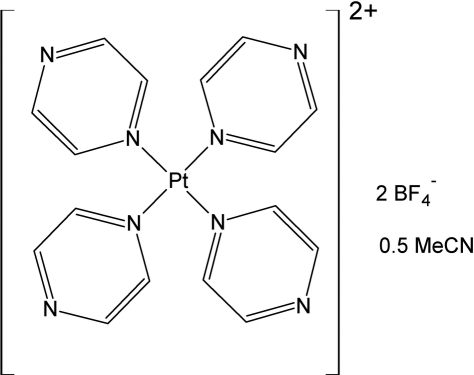


## Experimental

### 

#### Crystal data

[Pt(C_4_H_4_N_2_)_4_](BF_4_)_2_·0.5C_2_H_3_N
*M* *_r_* = 709.6
Monoclinic, 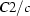

*a* = 13.862 (4) Å
*b* = 10.819 (3) Å
*c* = 17.262 (5) Å
β = 91.607 (3)°
*V* = 2587.8 (13) Å^3^
*Z* = 4
Mo *K*α radiation
μ = 5.50 mm^−1^
*T* = 296 (2) K
0.27 × 0.21 × 0.19 mm

#### Data collection

Bruker APEXII CCD diffractometer
Absorption correction: multi-scan (*SADABS*; Bruker, 2004 ▶) *T*_min_ = 0.246, *T*_max_ = 0.352
11212 measured reflections
2736 independent reflections
1846 reflections with *I* > 2σ(*I*)
*R*_int_ = 0.039

#### Refinement

*R*[*F* ^2^ > 2σ(*F* ^2^)] = 0.033
*wR*(*F* ^2^) = 0.114
*S* = 1.20
2736 reflections
167 parameters
2 restraints
H-atom parameters constrained
Δρ_max_ = 1.58 e Å^−3^
Δρ_min_ = −0.45 e Å^−3^

### 

Data collection: *APEX2* (Bruker, 2006 ▶); cell refinement: *APEX2*; data reduction: *APEX2*; program(s) used to solve structure: *SHELXS97* (Sheldrick, 2008 ▶); program(s) used to refine structure: *SHELXL97* (Sheldrick, 2008 ▶); molecular graphics: *XP* in *SHELXTL* (Sheldrick, 2008 ▶) and *PLATON* (Spek, 2003 ▶); software used to prepare material for publication: *SHELXL97* and *WinGX* (Farrugia, 1999 ▶).

## Supplementary Material

Crystal structure: contains datablocks global, I. DOI: 10.1107/S1600536808033679/pk2119sup1.cif

Structure factors: contains datablocks I. DOI: 10.1107/S1600536808033679/pk2119Isup2.hkl

Additional supplementary materials:  crystallographic information; 3D view; checkCIF report

## Figures and Tables

**Fig. 1.**
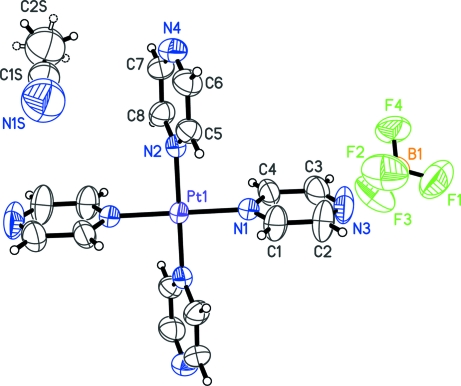
Ellipsoid plot of tetrapyrazineplatinum(II) tetrafluoroborate. Unlabelled atoms are generated via inversion through Pt1, symmetry code: -x, -y + 1, -z (50% probability displacement ellipsoids).

**Fig. 2.**
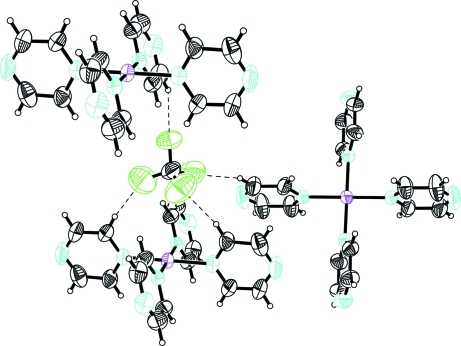
View of the hydrogen bonding between the tetrafluoroborate anion and the tetrapyrazineplatinum(II) cation (50% probability displacement ellipsoids).

## supplementary crystallographic information

### Comment

The synthesis of the nitrate salt of tetrapyrazineplatinum(II), which has use as a precursor to other platinum compounds, was recently reported in low yield (Klika *et al.*, 2007). In our pursuit of utilizing pyrazine as a bridging ligand to form compounds which add to the growing number of supramolecular metallacycles such as those by Schweiger *et al.* (2001), Willermann *et al.* (2006), and Derossi *et al.* (2007), to name but a few, we sought a more favorable yield for this tetrapyrazineplatinum(II) precursor. We settled on a synthetic method that generates a high yield of tetrapyrazineplatinum(II) tetrafluoroborate following the use of a large excess of pyrazine and nitromethane, a non-coordinating solvent which Pearson *et al.* (1960) reported as increasing the lability of a ligand bound to Pt^II^.

The structure of the cation of tetrapyrazineplatinum(II) tetrafluoroborate (Figure 1) has a square planar conformation. The Pt^II^ ion resides on an inversion center with Pt1—N1 and Pt1—N2 distances of 2.026 (6) and 2.012 (5) Å, respectively. The two unique coordinated pyrazine ligands (N1 C1 C2 C3 C4 N3, N2 C5 C6 C7 C8 N4) are slightly canted relative to the (N1—Pt—N2) plane (dihedral angles between pyrazine planes and N1—Pt1—N2 plane are 84.1 (3)° and 69.3 (3)°, respectively). The canted conformation of the pyrazine ligands around the platinum atom is similar to that of pyridine in the closely-related tetrapyridine platinum(II) chloride trihydrate reported by Wei *et al.* (1989).

The tetrafluoroborate anion is positioned near three tetrapyrazineplatinum(II) cations and oriented such that every flourine atom is between 2.1 and 2.4Å from a hydrogen atom in the 3 or 5 position of a coordinated pyrazine. (Figure 2).

### Experimental

53 mg (0.099 mmol) of [Pt(NCMe)_4_](BF_4_)_2_, (synthesized according to Wendt *et al.* (1997)) and 344 mg (4.3 mmol) of pyrazine (Aldrich) were dissolved in 11 ml of degassed MeNO_2_. The schlenk flask was covered in foil and the stirring solution was moderately heated under a nitrogen environment for 18 h. 50 ml of degassed Et_2_O was added, the solution was cooled in an ice bath, and the resulting white solid was isolated by removal of the liquid *via* cannula and washing with 2x5mL degassed Et_2_O. The resulting solid was vacuum dried to give 67 mg (98% yield.) of a white solid. FT—IR (nujol, CsI plates): (cm^-1^) 1433(*s*,*C*—H), 1055 (*b*,B—F), 520 and 503 (*w*, Pt—N). ^1^H NMR (d-MeNO_2_): 9.00 (*m*) and 8.79 (*m*). ESI-*M*. S. 602 (Pt(C_4_H_4_N_2_)_4_)(BF_4_)^+^), 258 ([Pt(C_4_H_4_N_2_)_4_]^2+^).

Pale yellow crystals were grown by liquid diffusion of diethylether into a nitromethane solution containing tetrapyrazineplatinum(II) tetrafluoroborate and excess pyrazine.

### Refinement

The positions of the pyrazine and methyl H atoms were refined using a riding model, in accordance with the HFIX 43 and HFIX 137 instructions of SHELXL97, with pyrazine C—H distances of 0.93 Å, methyl C—H distance of 0.96 Å, and with *U*_iso_(H) values of 1.2Ueq(C) for pyrazine and 1.5Ueq(C) for methyl group in the acetonitrile solvent molecule. The acetonitrile solvate resides on a crystallographic two-fold axis with the methyl H atoms disordered in two position. Each of the methyl H atoms were refined accordingly as half occupied. The largest positive and negative peaks in the final difference map are 1.58 and -0.45 Å, respectively, from the Pt atom.

### Crystal data

**Table:**

[Pt(C_4_H_4_N_2_)_4_](BF_4_)_2_·0.5C_2_H_3_N	*F*(000) = 1356
*M**_r_* = 709.6	*D*_x_ = 1.821 Mg m^−^^3^
Monoclinic, *C*2/*c*	Mo *K*α radiation, λ = 0.71073 Å
Hall symbol: -C 2yc	Cell parameters from 8501 reflections
*a* = 13.862 (4) Å	θ = 2.3–27.8°
*b* = 10.819 (3) Å	µ = 5.50 mm^−^^1^
*c* = 17.262 (5) Å	*T* = 296 K
β = 91.607 (3)°	Block, pale yellow
*V* = 2587.8 (13) Å^3^	0.27 × 0.21 × 0.19 mm
*Z* = 4	

### Data collection

**Table:**

Bruker APEXII CCD diffractometer	1846 reflections with *I* > 2σ(*I*)
ω scans	*R*_int_ = 0.039
Absorption correction: multi-scan (SADABS; Bruker, 2004)	θ_max_ = 26.7°, θ_min_ = 2.4°
*T*_min_ = 0.246, *T*_max_ = 0.352	*h* = −17→17
11212 measured reflections	*k* = −13→13
2736 independent reflections	*l* = −21→21

### Refinement

**Table:**

Refinement on *F*^2^	2 restraints
Least-squares matrix: full	H-atom parameters constrained
*R*[*F*^2^ > 2σ(*F*^2^)] = 0.033	*w* = 1/[σ^2^(*F*_o_^2^) + (0.0518*P*)^2^ + 10.4546*P*] where *P* = (*F*_o_^2^ + 2*F*_c_^2^)/3
*wR*(*F*^2^) = 0.114	(Δ/σ)_max_ = 0.012
*S* = 1.20	Δρ_max_ = 1.58 e Å^−^^3^
2736 reflections	Δρ_min_ = −0.45 e Å^−^^3^
167 parameters	

### Special details

**Table:**

Geometry. All e.s.d.'s (except the e.s.d. in the dihedral angle between two l.s. planes) are estimated using the full covariance matrix. The cell e.s.d.'s are taken into account individually in the estimation of e.s.d.'s in distances, angles and torsion angles; correlations between e.s.d.'s in cell parameters are only used when they are defined by crystal symmetry. An approximate (isotropic) treatment of cell e.s.d.'s is used for estimating e.s.d.'s involving l.s. planes.

### Fractional atomic coordinates and isotropic or equivalent isotropic displacement parameters (Å^2^)

**Table:**

	*x*	*y*	*z*	*U*_iso_*/*U*_eq_	Occ. (<1)
Pt1	0	0.5	0	0.04102 (14)	
N1	0.0732 (4)	0.5078 (5)	0.1031 (4)	0.0463 (13)	
N2	0.1147 (4)	0.4171 (5)	−0.0466 (3)	0.0435 (13)	
N3	0.1705 (8)	0.5173 (9)	0.2459 (5)	0.090 (3)	
N4	0.2743 (5)	0.3006 (7)	−0.1078 (4)	0.0693 (19)	
C1	0.1234 (7)	0.6071 (9)	0.1267 (5)	0.072 (3)	
H1A	0.1267	0.6756	0.0943	0.087*	
C2	0.1699 (8)	0.6096 (12)	0.1971 (6)	0.092 (3)	
H2A	0.2031	0.6811	0.2113	0.11*	
C3	0.1246 (7)	0.4181 (12)	0.2223 (5)	0.081 (3)	
H3A	0.1257	0.3491	0.2545	0.097*	
C4	0.0729 (6)	0.4100 (8)	0.1496 (5)	0.060 (2)	
H4A	0.04	0.3383	0.1352	0.072*	
C5	0.1983 (6)	0.4785 (6)	−0.0567 (5)	0.0509 (19)	
H5A	0.2027	0.5615	−0.043	0.061*	
C6	0.2764 (6)	0.4203 (9)	−0.0868 (5)	0.066 (2)	
H6A	0.3329	0.465	−0.093	0.079*	
C7	0.1921 (7)	0.2422 (8)	−0.0975 (5)	0.063 (2)	
H7A	0.1881	0.1592	−0.1112	0.076*	
C8	0.1128 (6)	0.2975 (7)	−0.0678 (5)	0.058 (2)	
H8A	0.0566	0.2518	−0.0622	0.07*	
B1	0.1048 (10)	0.1540 (11)	0.4316 (8)	0.081 (3)	
F1	0.1311 (10)	0.1855 (9)	0.5039 (6)	0.226 (6)	
F2	0.1324 (7)	0.2427 (8)	0.3805 (6)	0.163 (4)	
F3	0.0102 (6)	0.1483 (9)	0.4290 (6)	0.172 (4)	
F4	0.1480 (6)	0.0465 (7)	0.4153 (5)	0.125 (3)	
N1S	0	0.224 (3)	−0.25	0.174 (15)*	0.5
C1S	0	0.124 (3)	−0.25	0.093 (9)*	0.5
C2S	0	−0.014 (3)	−0.25	0.16 (2)*	0.5
H2S1	0.039	−0.0435	−0.207	0.241*	0.25
H2S2	0.0259	−0.0435	−0.2976	0.241*	0.25
H2S3	−0.0649	−0.0435	−0.2454	0.241*	0.25

### Atomic displacement parameters (Å^2^)

**Table:**

	*U*^11^	*U*^22^	*U*^33^	*U*^12^	*U*^13^	*U*^23^
Pt1	0.0407 (2)	0.0471 (2)	0.0350 (2)	0.00181 (19)	−0.00349 (13)	−0.00503 (18)
N1	0.045 (3)	0.053 (3)	0.041 (3)	0.007 (3)	−0.003 (2)	−0.006 (3)
N2	0.045 (3)	0.049 (3)	0.037 (3)	0.004 (3)	−0.003 (2)	0.002 (2)
N3	0.092 (7)	0.128 (9)	0.049 (5)	−0.012 (5)	−0.026 (4)	−0.011 (5)
N4	0.054 (4)	0.081 (5)	0.073 (5)	0.015 (4)	0.003 (3)	−0.014 (4)
C1	0.082 (6)	0.074 (6)	0.060 (5)	−0.027 (5)	−0.016 (5)	−0.004 (4)
C2	0.101 (8)	0.111 (9)	0.062 (6)	−0.038 (7)	−0.028 (6)	−0.007 (6)
C3	0.080 (6)	0.108 (9)	0.053 (5)	0.021 (6)	−0.004 (5)	0.013 (6)
C4	0.063 (5)	0.062 (5)	0.055 (5)	0.006 (4)	−0.016 (4)	0.004 (4)
C5	0.047 (4)	0.047 (5)	0.058 (5)	0.000 (3)	−0.001 (3)	−0.001 (3)
C6	0.046 (4)	0.080 (6)	0.072 (6)	0.002 (4)	0.005 (4)	0.001 (5)
C7	0.074 (6)	0.049 (5)	0.066 (5)	0.016 (4)	−0.004 (4)	−0.013 (4)
C8	0.061 (5)	0.052 (4)	0.062 (5)	−0.004 (4)	0.000 (4)	−0.012 (4)
B1	0.091 (9)	0.064 (7)	0.088 (9)	0.024 (6)	−0.018 (7)	−0.003 (6)
F1	0.329 (15)	0.179 (9)	0.166 (9)	0.089 (9)	−0.080 (10)	−0.091 (8)
F2	0.192 (9)	0.109 (6)	0.188 (9)	0.034 (6)	0.032 (7)	0.062 (6)
F3	0.088 (5)	0.200 (9)	0.227 (11)	0.021 (6)	−0.015 (6)	0.031 (7)
F4	0.172 (8)	0.073 (3)	0.131 (6)	0.050 (5)	−0.001 (5)	−0.006 (4)

### Geometric parameters (Å, °)

**Table:**

Pt1—N2^i^	2.012 (5)	C4—H4A	0.93
Pt1—N2	2.012 (5)	C5—C6	1.368 (11)
Pt1—N1^i^	2.026 (6)	C5—H5A	0.93
Pt1—N1	2.026 (6)	C6—H6A	0.93
N1—C4	1.327 (10)	C7—C8	1.364 (11)
N1—C1	1.337 (10)	C7—H7A	0.93
N2—C8	1.345 (9)	C8—H8A	0.93
N2—C5	1.352 (9)	B1—F3	1.312 (14)
N3—C3	1.307 (14)	B1—F1	1.335 (14)
N3—C2	1.307 (13)	B1—F4	1.342 (12)
N4—C7	1.319 (11)	B1—F2	1.365 (14)
N4—C6	1.344 (11)	N1S—C1S	1.086 (14)
C1—C2	1.360 (12)	C1S—C2S	1.492 (10)
C1—H1A	0.93	C2S—H2S1	0.96
C2—H2A	0.93	C2S—H2S2	0.96
C3—C4	1.432 (12)	C2S—H2S3	0.96
C3—H3A	0.93		
			
N2^i^—Pt1—N2	180.0 (3)	N2—C5—C6	120.9 (7)
N2^i^—Pt1—N1^i^	89.3 (2)	N2—C5—H5A	119.6
N2—Pt1—N1^i^	90.7 (2)	C6—C5—H5A	119.6
N2^i^—Pt1—N1	90.7 (2)	N4—C6—C5	122.3 (8)
N2—Pt1—N1	89.3 (2)	N4—C6—H6A	118.8
N1^i^—Pt1—N1	180.00 (18)	C5—C6—H6A	118.8
C4—N1—C1	117.9 (7)	N4—C7—C8	123.3 (8)
C4—N1—Pt1	119.2 (5)	N4—C7—H7A	118.4
C1—N1—Pt1	123.0 (5)	C8—C7—H7A	118.4
C8—N2—C5	116.6 (6)	N2—C8—C7	121.0 (8)
C8—N2—Pt1	121.9 (5)	N2—C8—H8A	119.5
C5—N2—Pt1	121.5 (5)	C7—C8—H8A	119.5
C3—N3—C2	115.6 (9)	F3—B1—F1	106.8 (12)
C7—N4—C6	115.9 (7)	F3—B1—F4	113.9 (12)
N1—C1—C2	121.1 (9)	F1—B1—F4	107.8 (10)
N1—C1—H1A	119.4	F3—B1—F2	108.0 (10)
C2—C1—H1A	119.4	F1—B1—F2	110.5 (12)
N3—C2—C1	123.8 (10)	F4—B1—F2	109.8 (11)
N3—C2—H2A	118.1	N1S—C1S—C2S	180.000 (5)
C1—C2—H2A	118.1	C1S—C2S—H2S1	109.5
N3—C3—C4	123.4 (10)	C1S—C2S—H2S2	109.5
N3—C3—H3A	118.3	H2S1—C2S—H2S2	109.5
C4—C3—H3A	118.3	C1S—C2S—H2S3	109.5
N1—C4—C3	118.1 (8)	H2S1—C2S—H2S3	109.5
N1—C4—H4A	120.9	H2S2—C2S—H2S3	109.5
C3—C4—H4A	120.9		

Symmetry codes: (i) −*x*, −*y*+1, −*z*.
